# Early-Onset Colorectal Cancer: Are Neuroendocrine Tumors or Adenocarcinomas the Culprit? Analysis of the Largest U.S. Cancer Incidence Database, 2001–2020

**DOI:** 10.3390/jcm13041098

**Published:** 2024-02-15

**Authors:** Yazan Abboud, Madison Fraser, Imran Qureshi, Kaveh Hajifathalian

**Affiliations:** 1 Department of Internal Medicine, Rutgers New Jersey Medical School, Newark, NJ 07013, USA; ya296@njms.rutgers.edu (Y.A.); mf1124@njms.rutgers.edu (M.F.); iaq5@njms.rutgers.edu (I.Q.); 2 Department of Gastroenterology and Hepatology, Rutgers New Jersey Medical School, Newark, NJ 07103, USA

**Keywords:** early-onset colorectal cancer, incidence, adenocarcinoma, neuroendocrine tumors, epidemiology

## Abstract

(1) Background: While prior data showed an increasing incidence of colorectal cancer (CRC) in young adults, the contribution of adenocarcinoma (ADC) and neuroendocrine tumors (NETs) to this trend is not well studied. Therefore, we conducted a comparative analysis of the incidence rates and time trends of colorectal ADC and NETs in young adults (aged 24–54) using the United States Cancer Statistics (USCS) database. (2) Methods: Age-adjusted CRC incidence rates between 2001 and 2020 were calculated and categorized by sex, histopathology, and stage at diagnosis. Annual percentage change (APC) and average APC (AAPC) were computed via joinpoint regression utilizing weighted Bayesian information criteria to generate the simplest trend. Pairwise comparative analysis of ADC and NETs was conducted using tests of identicalness and parallelism. (3) Results: In this study, 514,875 patients were diagnosed with early-onset-CRC between 2001 and 2020 (54.8% men). While CRC incidence was significantly increased, including both ADC (448,670 patients) and NETs (36,205 patients), a significantly greater increase was seen for NETs (AAPC = 2.65) compared to ADC (AAPC = 0.91), with AAPC difference = 1.73 (*p* = 0.01) and non-identical non-parallel trends (*p*-values < 0.001). This was most notable in males (AAPC difference = 1.81, *p* = 0.03) and for early-stage tumors (AAPC difference = 3.56, *p* < 0.001). (4) Conclusions: Our study, covering ~98% of the U.S. population provides the first comparative analysis of early-onset CRC histopathological subtypes, showing that the rate of increase of NETs in young adults is much greater than that of ADC. Given that patients with NETs with malignant behavior can experience significant mortality, our findings are importance, highlighting the rapidly increasing NET incidence in young adults and encouraging early screening that can improve outcomes.

## 1. Introduction

Colorectal cancer (CRC) has been reported as the third most common and second most fatal cancer worldwide [1]. According to GLOBOCAN data, there were close to 2 million new cases and 1 million deaths related to CRC, with approximately half of those deaths occurring at the age of 50–74 years, in 2020 [2]. CRC can be divided into different subtypes based on their histological features. The vast majority of CRC are adenocarcinoma (ADC), with data showing a proportion as high as 96% of all CRC fitting into that subtype [3].

Though the incidence of CRC has been on the rise globally, there has been a substantial decline in the United States (U.S.) [4,5,6]. This can be partially attributed to changes in the exposure to risk factors but is largely due to the screening protocols in place [7]. There has, however, been an increase in the incidence of CRC among young adults in the U.S., which prompted the United States Preventive Services Task Force (USPSTF) to update their guidelines in 2021 to include individuals aged between 45 and 49 years as candidates for CRC screening [8,9].

Early-onset CRC accounts for approximately 10% of newly diagnosed CRC and is predicted to more than double in incidence by 2030 [10,11]. Interestingly, half of the cases diagnosed with early-onset CRC are sporadic, without associated hereditary syndromes or familial CRC [12]. There is increasing evidence to suggest that early-onset CRC is distinct from late-onset CRC, not only in terms of age of presentation but also in terms of clinical manifestation, location, and histopathological characteristics [13,14]. This has prompted researchers to consider early-onset CRC as a unique entity, with a gap in the literature as to the histopathological subtype that may be contributing to its rising incidence [15,16].

Simultaneously, there has been an increase in the incidence of colorectal neuroendocrine tumors (NETs), a CRC subtype that is typically considered rare [17,18,19]. NETs, previously known as carcinoid tumors, account for less than 1% of all CRCs, and their risk factors, management, and outcomes are distinct from those of ADC [19]. Not only does the rising incidence of NETs dictate the management of CRC, since the management of NETs is different from that of adenocarcinomas, but also it has implications regarding screening protocols, as the incidence of early-onset CRC continues to rise [20]. While prior data showed an increasing incidence of both ADC and NETs in younger adults aged 20–54 years [9], there is still a lack of research exploring the incidence of NETs compared to that of ADC in the context of early-onset CRC. Therefore, the aim of this study was to conduct a comprehensive comparative time trend analysis of the incidence rates and trends of ADC and NETs, in young adults, using the most comprehensive U.S. cancer database, i.e., the United States Cancer Statistics (USCS) database.

## 2. Materials and Methods

This study is a nationwide time trend analysis of the incidence rates of early-onset CRC in the U.S. between 2001 and 2020. The USCS database was utilized to obtain the data. The data used in this study are all publicly available; they were de-identified and therefore were exempted from review by the institutional review board, based on the National Human Research Protections Advisory Committee policy.

The incidence rates of early-onset CRC between 1 January 2001 and 31 December 2020 were obtained from the USCS database, a comprehensive database of cancer incidence data in the U.S., which approximately covers 98% of the U.S. population [21]. The data in the USCS database include data from the Centers for Disease Control and Prevention (CDC) National Program of Cancer Registries (NPCR) and the National Cancer Institute (NCI) Surveillance, Epidemiology, and End Results (SEER) program. The USCS data are from all 50 states, the District of Columbia, and Puerto Rico [21]. To ensure high-quality data and maintain validation, the data collected in the U.S. cancer registries are exported into software programs and undergo standardization of the coding per the North American Association of Central Cancer Registries’ data standards [22]. 

The early-onset CRC incidence rate was defined as the number of patients aged 20–54 years who were diagnosed with CRC per 100,000 population each calendar year, as in prior studies [9,23]. Time trends were reported as annual percentage change (APC), which was defined as the percentage change in the incidence rates between two years, and average APC (AAPC), which was defined as the average percentage change in the incidence rates over the entire study period of 2001–2020. If the AAPC was statistically significant, the trend was defined as increasing or decreasing depending on the AAPC value; however, if the AAPC was not statistically significant, then the trend was defined as stable. The location of the tumor was specified as “primary site” with the codes C18.0 (cecum), C18.2 (ascending colon), C18.3 (hepatic flexure of the colon), C18.4 (transverse colon), C18.5 (splenic flexure of the colon), C18.6 (descending colon), C18.7 (sigmoid colon), C18.8 (overlapping lesion of the colon), C18.9 (colon, non-specified), C19.9 (rectosigmoid junction), and C20.9 (rectum, non-specified), with malignant behavior. The International Classification of Diseases for Oncology, Third Edition, Site Record ICD-O-3/WHO 2008 was used to identify ADC codes as follows: 8140, 8141, 8143, 8144, 8210, 8211, 8213, 8220, 8221, 8255, 8260–8263, 8310, 8323, 8440, 8460, 8470, 8472, 8480–8482, 8570, 8574, 8576. The NET codes were as follows: 8013, 8240, 8241, 8243, 8244, 8245, 8246, and 8249, as previously reported [9]. The tumor stage at diagnosis was defined as early stage (in situ and localized tumors) and late stage (tumors with regional or distant site or node involvement).

The early-onset CRC incidence rates were calculated and then age-adjusted to the standard 2000 U.S. population utilizing SEER*Stat software (v.8.4.2, National Cancer Institute, NCI). The APC and AAPC were estimated using Joinpoint Regression Software (v.5.0.2, NCI) using the weighted Bayesian information criteria (BIC) method, a data-driven method to generate trends over time, which is generally preferred given its flexibility and optimal performance, on average, across different analytical scenarios [24,25,26]. Pairwise comparison was conducted between trends using the tests of parallelism and coincidence, and the absolute AAPC difference was evaluated using a Taylor series expansion [27]. A two-sided *p*-value cut-off of 0.05 was utilized for statistical significance. Further analysis was conducted after categorizing the tumors by sex and stage at diagnosis. Lastly, a sensitivity analysis was conducted using microscopically confirmed early-onset CRC cases only.

## 3. Results

### 3.1. Early-Onset CRC Incidence Stratified by Histopathology

**Figure 1:**
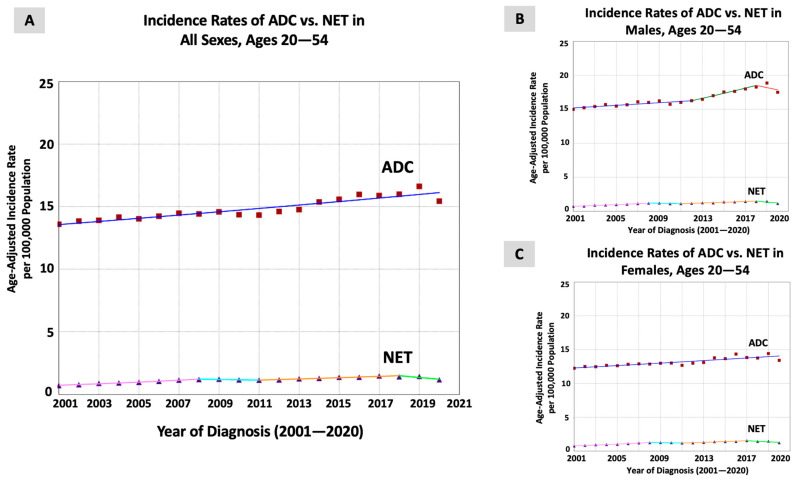
Time trends and age-adjusted incidence rates per 100,000 population for colorectal cancer (CRC) in young adults Aged 20–54 years categorized by histopathological subtype and sex (adenocarcinoma, ADC, and neuroendocrine tumors NETs).

There were 514,875 patients who were diagnosed with early-onset CRC between 2001 and 2020 (54.8% men) in the USCS database. While CRC incidence, including both ADC (448,670 patients; 87.1% of all cases) and NET (36,205 patients; 7.0% of all cases) subtypes, significantly increased in the examined period, there was a significant difference between the incidence trends of the two subtypes. The incidence rate of ADC appeared to significantly increase at a steady pace throughout the study period (APC = AAPC = 0.91, *p* < 0.001). For NETs, the incidence rate significantly increased between 2001 and 2008 (APC = 7.32, *p* < 0.001), became stable between 2008 and 2011 (APC = −1.98, *p* = 0.52), significantly increased between 2011 and 2018 (APC = 3.85, *p* < 0.001), and significantly decreased between 2018 and 2020 (APC = −9.62, *p* = 0.008). When evaluating the trends over the entire period, a significantly greater increase was seen for NETs (AAPC = 2.65, *p* < 0.001) compared to ADC (AAPC = 0.91, *p* < 0.001), with an absolute AAPC difference of 1.73, *p* = 0.01 (Table 1 and Figure 1A). The trends were non-identical and non-parallel (*p*-values < 0.001), suggesting that the incidence rate of NETs was distinct from and increased at a significantly greater pace compared to that of ADC.

### 3.2. Early-Onset CRC Incidence Stratified by Histopathology and Sex

When evaluating the trends in males (264,978 patients; 51.5% of all cases), we found that the incidence rate of ADC significantly increased between 2001 and 2012 (APC = 0.61, *p* < 0.001) and between 2012 and 2018 (APC = 2.19, *p* < 0.001), but was stable between 2018 and 2020 (APC = −1.78, *p* = 0.32). For NETs, the incidence rate significantly increased between 2001 and 2008 (APC = 7.32, *p* < 0.001), was stable between 2008 and 2011 (APC = −2.23, *p* = 0.57), significantly increased between 2011 and 2018 (APC = 4.21, *p* < 0.001), and significantly decreased between 2018 and 2020 (APC = −10.23, *p* = 0.02). When assessing the trends over the entire period, similar results were obtained, with the incidence rate of NETs (AAPC = 2.67, *p* = 0.001) increasing faster compared to that of ADC (AAPC = 0.85, *p* < 0.001), showing an absolute AAPC difference of 1.81, *p* = 0.03 (Table 1 and Figure 1B).

In females (219,897 patients; 42.7% of all cases), the incidence of ADC significantly increased at a steady pace through the entire study period (APC = AAPC = 0.71, *p* < 0.001). For NETs, the incidence rate significantly increased between 2001 and 2008 (APC = 7.37, *p* < 0.001), was stable between 2008 and 2011 (APC = −2.26), significantly increased between 2011 and 2017 (APC = 4.31, *p* = 0.006), and was stable between 2017 and 2020 (APC = −5.45, *p* = 0.06). When assessing the trends over the entire period, there was no significant difference in the rate of increase between NETs (AAPC = 2.74, *p* = 0.01) and ADC (AAPC = 0.71, *p* < 0.001), with an absolute AAPC difference of 2.03, *p* = 0.06 (Table 1 and Figure 1C).

### 3.3. Early-Onset CRC Incidence Stratified by Histopathology and Stage at Diagnosis

**Figure 2:**
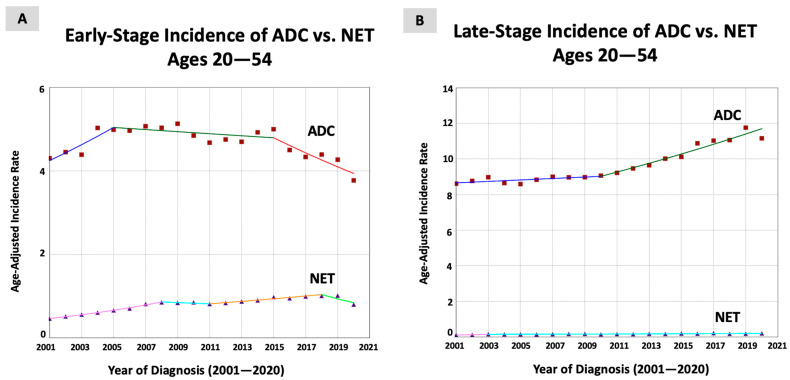
Time trends and age-adjusted incidence rates per 100,000 population for colorectal cancer (CRC) in young adults aged 20–54 years categorized by histopathological subtype and stage at diagnosis (adenocarcinoma, ADC, and neuroendocrine tumors, NETs).

When categorizing the tumors by stage at diagnosis, the trends varied. For early-stage tumors (168,975 patients; 32.8% of all cases), the incidence rate was found to increase only for NETs (AAPC = 3.17, *p* < 0.001) and not for ADC (AAPC = −0.39, *p* = 0.44), with a significant difference between the two trends (absolute AAPC difference of 3.56, *p* < 0.001). However, for late-stage tumors (294,966 patients; 57.3% of all cases), the incidence rate was shown to increase for both NETs (AAPC = 3.02, *p* = 0.02) and ADC (AAPC = 1.60, *p* < 0.001), without a significate difference (absolute AAPC difference= 1.43, *p* = 0.30, Table 2 and Figure 2).

### 3.4. Sensitivity Analysis

**Figure 3:**
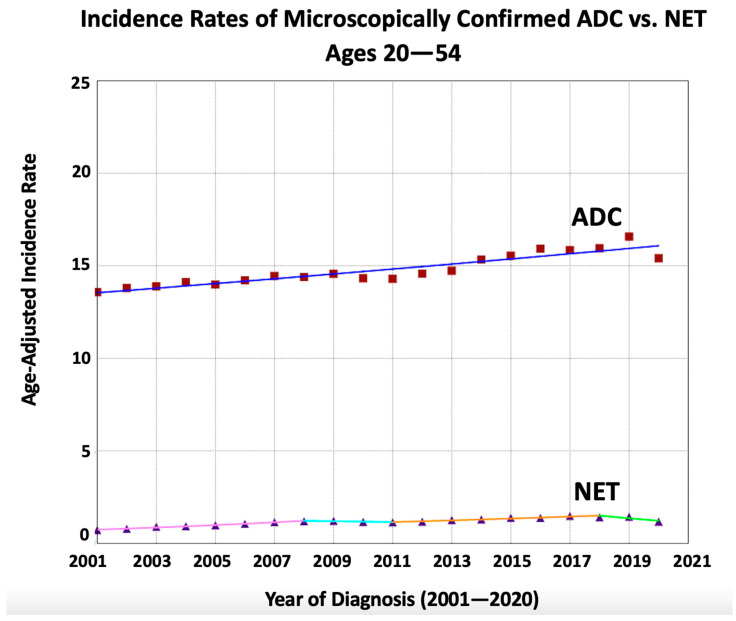
Time trends and age-adjusted incidence rates per 100,000 population for microscopically confirmed colorectal cancer (CRC) in young adults aged 20–54 years categorized by histopathological subtype (adenocarcinoma, ADC, and neuroendocrine tumors, NETs).

Sensitivity analysis including only microscopically confirmed cases of early-onset CRC (483,753 patients; 94.0% of all cases) showed similar results to those of the overall analysis including all modalities of diagnosis. The incidence rate of NETs was found to increase at a greater pace compared to that of ADC (AAPC = 2.63 vs. 0.91, absolute AAPC difference 1.72, *p* = 0.007) with non-identical non-parallel data (*p*-values < 0.001, Table 3 and Figure 3).

## 4. Discussion

Our study evaluating 514,875 patients with early-onset CRC in the U.S. (covering approximately 98% of the population) showed an increase in CRC incidence between 2001 and 2020. Comparative analysis of early-onset CRC histopathological subtypes showed that while NETs were overall less common compared to ADC, the incidence of NETs in young adults experienced a much steeper increase than that of ADC in males and early-stage tumors. However, the incidence rates of NETs and ADC increased similarly in females and late-stage tumors. We also showed that while the incidence of early-stage colorectal ADC was stable, that of late-stage colorectal ADC continued to experience a significant increase in young adults as we entered the 2020s.

Colorectal NETs have typically been examined separately from other CRC pathologies, and therefore there is limited literature evaluating NET incidence along with those of other CRC subtypes. NETs have been noted to represent 4–20% of colon cancer cases and 8–34% of rectal cases [9]; yet, many CRC incidence studies did not stratify the examined tumors based on histopathology. Studies that examined both ADC and NET incidence rates showed that both NET and ADC incidences were increasing in the U.S. In addition to supporting these conclusions, our data add to the literature by providing the analysis of a significantly larger sample over a recent time period (514,875 patients between 2001 and 2020) compared to prior studies (426,262 patients between 1992 and 2015 [28] and 119,624 patients between 2002 and 2016) [9]. The main results of our study and the aforementioned two studies are shown in Table 4. In addition, we reported the first comparative analysis of ADC and NETs, showing an increasing rate of NETs at a significantly greater pace compared to ADC, and confirmed this observation in microscopically diagnosed tumors. This has important implications for screening and research allocation regarding early-onset CRC, when considering which histopathologic subtypes may be increasing. While research on adenocarcinoma has seen advancements leading to improvements in patient mortality, patient mortality caused by colorectal neuroendocrine tumors is still high, and progress still slow, despite the increasing incidence of NETs in the young population [29]. A study from 2000–2011 by Shafqat et al. showed that patients with colorectal NETs had a much poorer median survival and 5-year survival across all age groups when compared to patients with high-grade adenocarcinoma (7.1 months versus 36 months and 16.3% versus 50.2%, respectively) [29]. This suggests a potential gap in knowledge and resources devoted to the treatment of colorectal NETs—an alarming postulate in the setting of early-onset colorectal NETs’ rising incidence. Likewise, the poor outcomes exhibited specifically by patients with colonic NETs (5-year survival of 62% in all stages) [30] further validate the need for earlier CRC screening. 

The incidence of CRC at all ages has historically been higher in males than in females, and early-onset CRC has recently been shown to follow the same trend (apart from early-onset distal CRC, which shows female predominance) [31,32]. We add to these existing data by further classifying early-onset histopathological subtype trends and their associations with sex. Interestingly, our data showed that early-onset colorectal NETs, when compared to ADC, displayed a steeper increase in incidence in males specifically (absolute AAPC difference = 1.81, *p* = 0.03), but similar rates of increase were seen in females (absolute AAPC difference = 2.03, *p* = 0.06). This suggests that NETs may be contributing to the previously reported overall increase in early-onset CRC in males. It should be noted that colorectal NETs typically display female predominance, as shown in prior studies [33]. However, we argue that with this rising incidence of early-onset colorectal NET cases compared to ADC in males, clinicians should maintain a degree of suspicion for NETs when screening or surveilling for CRC in young, male patients. 

Theoretically, early-onset colorectal NETs should benefit from the early-screening guidelines intended for ADC, given that colorectal NETs, like ADC, are also often relatively asymptomatic until late stages and found incidentally on colonoscopy. Our identification of increasing early-stage, early-onset NET rates supports this screening hypothesis benefit (AAPC = 3.17, *p* < 0.001). However, ADC would not appear to benefit from an early screening in the same way, given there was no observed increase in the incidence of early-onset, early-stage ADC in our analysis (AAPC = −0.39, *p* = 0.44). Contrarily, there continue to be increases in both late-stage, early-onset ADC (AAPC = 1.60, *p* < 0.001) and NETs (AAPC = 3.02, *p* = 0.02), as previous studies also reported [34]. Nevertheless, further exploring the impact of early screening on the incidence rates will be an important area of research in the future, especially with the newer guidelines recommending screening over the age of 45 years that have been implemented for adequate cause-and-effect observational time.

Increased detection and improvements in CRC diagnosis as described above may be contributing factors to the increasing colorectal NET incidence rates. However, the increasing rates of colorectal NETs may also be due to similar predisposing factors as observed for other gastrointestinal neuroendocrine tumors, such as increasing obesity and diabetes among young adults [35]. Additionally, the rates of early-onset rectal NETs. in particular, have shown an association with increased alcohol use and lower HDL levels [36], two independent risk factors that may support the connection between rising early-onset NETs and the obesity epidemic. Similar risk factors have already been proven for early-onset ADC development [37]. With that in mind, the non-increasing early-stage ADC incidence rate and the increasing late-stage ADC incidence rate in young adults argue against early screening alone as the reason behind these trends. Our data warrant further investigations of the effect on outcomes of a potential delay in diagnosing ADC in young adults.

When evaluating the variation in early-onset CRC incidence rates and time trends between different states and regions, limited data exist in the literature. A prior analysis by Siegel et al. showed an increase in early-onset CRC in 36 out of 47 states in the U.S. between 1995 and 2015 [38]. There was a variation in the incidence rates between the states by race. Furthermore, between 2011 and 2015, the lowest rates were seen in western states, and highest rates were seen in southern states [38]. Another analysis by Shah et al. evaluating early-onset CRC between 2001 and 2017 showed similar trends, with increasing incidence rates in most U.S. states, most notably seen in Utah, which had the highest AAPC [39]. When looking at international trends, prior studies showed an increasing trend of early-onset CRC incidence in 19 countries and a decreasing trend in three countries (Italy, Austria, and Lithuania [40]). As for the global colorectal NET trends, there has been a rising incidence in NETs, though studies have been limited by either the use of single-center data or a lack of adequate population coverage by registries/states [41]. Our study adds to the existing literature by comprehensively investigating the recent trends of early-onset colorectal ADC and NETs, showing increasing incidence of both histopathological subtypes. We also provide further insight into the epidemiology of those histopathological subtypes in the U.S., while categorizing the rates and trends by sex and stage at diagnosis. While it is hard to elucidate the exact causes of the variation seen between different states and regions, it may be related to disproportionate exposure to risk factors, including, but not limited to, geographical variation in the rate of change of the obesity rates. Moreover, it could also be driven by geographical differences in the rate of completing screening and surveillance colonoscopies, which can lead to increased detection of early-onset CRC [42].

Our study has several strengths. We provide the first comparative analysis of different histopathological subtypes of early-onset CRC using a comprehensive U.S. cancer database evaluating more than half a million cases over a recent time period. We also provide data on the contributions of sex and stage at diagnosis to the increasing incidence of early-onset CRC. Furthermore, our analysis was conducted utilizing joinpoint regression and modified BIC methods, which are statistical methodologies that are recommended to be used for large databases and have shown to be flexible across different analysis scenarios [25,43]. Lastly, we provide a sensitivity analysis of our findings using microscopically confirmed cases only and showing similar results to those of the overall analysis. However, our study suffers from several limitations such as its observational nature and a lack of variables to assess for risk factors for ADC and NETs. Moreover, other limitations that can arise from large databases include the loss of records and potential miscoding [44]. However, the USCS database is the most comprehensive database on cancer incidence data in the U.S. and undergoes several checks to maintain a high quality prior to data publication [21].

## 5. Conclusions

We provide the first comprehensive comparative analysis of early-onset CRC histopathological subtypes showing that the rate of increase in young adults diagnosed with NETs has been much steeper than in young adults diagnosed with ADC in the last years. Most of the prior literature did not account for NETs when evaluating early-onset CRC. Given that NETs with malignant behavior can lead to significant mortality, our findings are important, highlighting the rapidly increasing incidence of NETs in younger adults and encouraging the early detection and adherence to recent guidelines of screening at younger ages, which can improve outcomes. This is also of utmost importance, given our findings of an increasing incidence of late-stage ADC and not of early-stage tumors, suggesting delayed diagnosis in young adults. Our findings have public health implications and can guide healthcare policymakers to encourage early detection and advocate for an early screening of CRC. Future studies are needed to evaluate risk factors contributing to ADC and NETs in young adults with CRC in the U.S.

## Figures and Tables

**Table 1:** Early-onset colorectal cancer (CRC) incidence trends based on histopathological subtypes among different sex-specific groups.

Histopathological Subtype	Early-Onset CRC Cases (*N* = 514,875) ^a^	Trends ^b^			Sex-Specific AAPC Difference (95% CI) ^c^	Pairwise Comparison *p*-Values		
		Time Period	APC (95% CI)	AAPC (95% CI)		Sex-Specific AAPC Difference	Coincidence ^d^	Parallelism ^e^
Both Sexes								
ADC	448,670 (87.1%)	2001–2020	0.91 * (0.73 to 1.10)	0.91 * (0.73 to 1.10)	−1.73 * (−2.99 to −0.48)	0.01	<0.001	<0.001
NET	36,205 (7.0%)	2001–2008	7.32 * (6.19 to 8.47)	2.65 * (1.41 to 3.90)				
		2008–2011	−1.98 (−8.38 to 4.88)					
		2011–2018	3.85 * (2.72 to 4.99)					
		2018–2020	−9.62 * (−15.50 to −3.32)					
Males								
ADC	247,526 (48.1%)	2001–2012	0.61 * (0.34 to 0.89)	0.85 * (0.39 to 1.32)	−1.81 * (−3.49 to −0.13)	0.03	<0.001	<0.001
		2012–2018	2.19 * (1.31 to 3.07)					
		2018–2020	−1.78 (−5.47 to 2.05)					
NET	17,452 (3.4%)	2001–2008	7.32 * (5.85 to 8.81)	2.67 * (1.06 to 4.29)				
		2008–2011	−2.23 (−10.46 to 6.76)					
		2011–2018	4.21 * (2.75 to 5.70)					
		2018–2020	−10.23 * (−17.70 to −2.81)					
Females								
ADC	201,144 (39.1%)	2001–2020	0.71 * (0.52 to 0.90)	0.71 * (0.52 to 0.90)	−2.03 (−4.20 to 0.14)	0.06	<0.001	<0.001
NET	18,753 (3.6%)	2001–2008	7.37 * (5.32 to 9.45)	2.74 * (0.60 to 4.93)				
		2008–2011	−2.26 (−13.52 to 10.47)					
		2011–2017	4.31 * (1.55 to 7.14)					
		2017–2020	−5.45 (−11.08 to 0.54)					

ADC: adenocarcinoma, NETs: neuroendocrine tumors, APC: annual percentage change, AAPC: average annual percentage change.; ^a^ Data are presented as count numbers followed by percentages of the count numbers from the total cases of early-onset CRC in the database.; ^b^ Time trends were computed using Joinpoint Regression Program with 3 maximum joinpoints allowed (4-line segments). ^c^ A negative value indicates a greater AAPC in NETs compared to ADC. ^d^ Tests to evaluate whether the incidence trends were identical. A significant *p*-value indicates that the trends were not identical. ^e^ Tests to evaluate whether the incidence trends were parallel. A significant *p*-value indicates that the trends were not parallel. * Implies statistical significance.

**Table 2:** Early-onset colorectal cancer (CRC) incidence trends based on histopathological subtypes and stage at diagnosis.

Histopathological Subtype	Early-Onset CRC Cases (*N* = 514,875) ^a^	Trends ^b^			Sex-Specific AAPC Difference (95% CI) ^c^	Pairwise Comparison *p*-Values		
		Time Period	APC (95% CI)	AAPC (95% CI)		Sex-Specific AAPC Difference	Coincidence ^d^	Parallelism ^e^
Early Stage								
ADC	144,130 (28.0%)	2001–2005	4.37 * (0.78 to 8.10)	−0.39 (−1.40 to 0.62)	−3.56 * (−5.20 to −1.92)	<0.001	<0.001	<0.001
		2005–2015	−0.50 (−1.45 to 0.45)					
		2015–2020	−3.84 * (−6.31 to −1.31)					
NET	24,845 (4.8%)	2001–2008	9.18 * (7.95 to 10.41)	3.17 * (1.88 to 4.47)				
		2008–2011	−1.74 (−8.32 to 5.32)					
		2011–2018	3.48 * (2.31 to 4.67)					
		2018–2020	−9.92 * (−16.01 to −3.40)					
Late Stage								
ADC	289,826 (56.3%)	2001–2010	0.46 (−1.17 to 1.10)	1.60 * (1.23 to 1.97)	−1.43 (−4.14 to 1.29)	0.30	<0.001	0.41
		2010–2020	2.63 * (2.12 to 3.14)					
NET	5140 (1.0%)	2001–2003	15.15 (−11.38 to 49.63)	3.02 * (0.37 to 5.75)				
		2003–2020	1.68 * (0.93 to 2.45)					

ADC: adenocarcinoma, NETs: neuroendocrine tumors, APC: annual percentage change, AAPC: average annual percentage change. ^a^ Data are presented as count numbers followed by percentages of the count numbers from the total cases of early-onset CRC in the database. ^b^ Time trends were computed using Joinpoint Regression Program with 3 maximum joinpoints allowed (4-line segments). ^c^ A negative value indicates a greater AAPC in NETs compared to ADC. ^d^ Tests to evaluate whether the incidence trends were identical. A significant *p*-value indicates that the trends were not identical. ^e^ Tests to evaluate whether the incidence trends were parallel. A significant *p*-value indicates that the trends were not parallel. * Implies statistical significance.

**Table 3:** Microscopically confirmed cases of early-onset colorectal cancer (CRC) incidence trends based on histopathological subtypes.

Histopathological Subtype	Early-Onset CRC Cases (*N* = 514,875) ^a^	Trends ^b^			Sex-Specific AAPC Difference (95% CI) ^c^	Pairwise Comparison *p*-Values		
		Time Period	APC (95% CI)	AAPC (95% CI)		Sex-Specific AAPC Difference	Coincidence ^d^	Parallelism ^e^
Microscopically Confirmed Cases								
ADC	447,664 (86.9%)	2001–2020	0.91 * (0.73 to 1.09)	0.91 * (0.73 to 1.09)	−1.72 * (−2.98 to −0.46)	0.007	<0.001	<0.001
NET	36,089 (7.0%)	2001–2008	7.31 * (6.17 to 8.46)	2.63 * (1.39 to 3.89)				
		2008–2011	−1.94 (−8.39 to 4.95)					
		2011–2018	3.84 * (2.71 to 4.99)					
		2018–2020	−9.77 * (−15.68 to −3.44)					

ADC: adenocarcinoma, NETs: neuroendocrine tumors, APC: annual percentage change, AAPC: Average annual percentage change. ^a^ Data are presented as count numbers followed by percentages of the count numbers from the total cases of early-onset CRC in the database.; ^b^ Time trends were computed using Joinpoint Regression Program with 3 maximum joinpoints allowed (4-line segments). ^c^ A negative value indicates a greater AAPC in NETs compared to ADC.; ^d^ Tests to evaluate whether the incidence trends were identical. A significant *p*-value indicates that the trends were not identical. ^e^ Tests to evaluate whether the incidence trends were parallel. A significant *p*-value indicates that the trends were not parallel. * Implies statistical significance.

**Table 4:** Time trends of the incidence rates of early-onset colorectal cancer in the current study compared to those reported in prior literature.

Study	Current Study			Montminy et al. [9]			Lumsdaine et al. [28]		
**Database**	USCS (covers ~98% of U.S. population)			SEER 18 (covers ~27.8% of U.S. population)			SEER 13 (covers ~13.4% of U.S. population)		
**Time period**	2001–2020			2000–2016			1992–2015		
**Sample size**	514,875			119,624			426,262		
**APC: ADC** **(95% CI)**	Ages 20–54	2001–2020	0.91 * (0.73–1.09)	Ages 20–29	2000–2005	5.6 * (0.5–11.1)	Ages 20–44 #	1992–2015	2.4 * (1.9–2.8)
					2005–2016	−0.30 (−1.7–1.1)			
				Ages 30–39	2000–2016	1.6 * (1.2–2.0)			
				Ages 40–49		0.9 * (0.7–1.2)	Ages 45–54 #		1.0 * (0.7–1.3)
				Ages 50–54		0.2 (−0.1–0.5)			
**APC: NET** **(95% CI)**	Ages 20–54	2001–2008	7.31 * (6.17–8.46)	Ages 20–29	2000–2016	4.3 * (1.9–6.8)	Ages 20–44 #	1992–1995	−13 (−26.9–3.6)
		2008–2011	−1.94 (−8.39–4.95)	Ages 30–39		2.4 * (1.0–4.0)		2001–2015	2.2 * (0.5–2.8)
		2011–2018	3.84 * (2.71–4.99)	Ages 40–49		2.5 * (1.4–3.6)	Ages 45–54 #	1992–1994	−6.1 (−25.7–18.7)
		2018–2020	−9.77 * (−15.68–−3.44)	Ages 50–54	2000–2007	2.4 * (0.4–4.4)		1994–2007	10.6 * (9.1–12.2)
					2007–2016	10.6 * (6.2–15.2)		2007–2015	2.2 (−0.3–4.8)
**AAPC: ADC** **(95% CI)**	Ages 20–54	2001–2020	0.91 * (0.73–1.09)	Ages 20–29	2000–2016	Not calculated/provided	Ages 20–44 #	1992–2015	2.4 * (1.9–2.8)
				Ages 30–39		1.6 * (1.2–2.0)			
				Ages 40–49		0.9 * (0.7–1.2)	Ages 45–54 #		1.0 * (0.7–1.3)
				Ages 50–54		0.2 (−0.1–0.5)			
**AAPC: NET** **(95% CI)**	Ages 20–54	2001–2020	2.63 * (1.39–3.89)	Ages 20–29	2000–2016	4.3 * (1.9–6.8)	Ages 20–44 #	1992–2015	2.9 (−0.1–6.0)
				Ages 30–39		2.4 * (1.0–4.0)			
				Ages 40–49		2.5 * (1.4–3.6)	Ages 45–54 #		6.1 * (3.8–8.4)
				Ages 50–54		Not calculated/provided			

* Indicates statistical significance. # Calculated APC/AAPC for rectal ADC and NETs only, proximal and distal colon APCs not performed/provided. USCS: United States Cancer Statistics, SEER: Surveillance Epidemiology and End Results, APC: annual percentage change, AAPC: average annual percentage change.

## Data Availability

Data used in this study can be obtained from the USCS database.
